# Varicella Zoster Virus Infection and Pregnancy: An Optimal Management Approach

**DOI:** 10.3390/pathogens14020151

**Published:** 2025-02-05

**Authors:** Ana Ion, Olguța Anca Orzan, Beatrice Bălăceanu-Gurău

**Affiliations:** 1 Faculty of Medicine, ‘Carol Davila’ University of Medicine and Pharmacy, 020021 Bucharest, Romania; ana.ion@rez.umfcd.ro (A.I.); beatrice.balaceanu@drd.umfcd.ro (B.B.-G.); 2 Department of Dermatology, ‘Elias’ University Emergency Hospital, 011461 Bucharest, Romania

**Keywords:** varicella, herpes zoster, pregnancy, infection, varicella-zoster virus

## Abstract

Varicella-zoster virus is an α-herpes virus with a double-stranded DNA genome, which causes two main clinical pictures: varicella or chickenpox and herpes zoster. Chickenpox is the primary infection, predominantly affecting children, and it presents with fever and a cutaneous eruption consisting of a vesicular, pruritic, and painful rash. Herpes zoster is a viral infection that typically develops in adulthood as a result of the reactivation of the varicella-zoster virus. If acquired during pregnancy, chickenpox may be responsible for serious complications for the mother, the fetus, or the newborn. The most frequent complication of primary varicella-zoster virus infection in mothers is varicella pneumonia, while encephalitis and hepatitis are rare. The effects on the fetus due to chickenpox infection depend on the stage of pregnancy when the mother becomes infected. If the infection occurs during the first trimester, it does not increase the risk of miscarriage. However, if the infection occurs during the first or second trimester, it may cause fetal varicella syndrome or congenital varicella syndrome. During pregnancy, if the varicella-zoster virus reactivates, it usually does not cause harm to the fetus or lead to any birth defects. However, it may increase maternal morbidity due to herpes zoster and its complications. In the case of primary varicella-zoster virus infection in pregnant women, about 20% of newborns may get neonatal or infantile herpes zoster without any complications. However, it is recommended to start early treatment of herpes zoster in pregnant women as it is believed to accelerate the healing process of skin lesions and alleviate pain, reducing both its duration and severity. Through this narrative review, we discuss the approach to the optimal management of varicella-zoster virus infection during pregnancy.

## 1. Introduction

The varicella-zoster virus (VZV) is a neurotropic α-herpesvirus characterized by a double-stranded DNA genome, and it is responsible for two distinct clinical conditions: varicella (chickenpox) and herpes zoster (HZ) (shingles) [1,2]. Varicella, the primary infection, predominantly affects children and presents with fever and a characteristic skin rash [1,3]. This rash consists of clusters of vesicular, pruritic, and occasionally painful lesions [1,3]. Following the initial infection, VZV establishes latency in the sensory nerve root ganglia, where it can persist in a dormant state for years. HZ results from the reactivation of VZV, typically triggered by immunosuppression. Reactivated viral particles travel along the affected nerve, producing a painful, vesicular rash localized to a specific dermatome—the area of skin innervated by the infected sensory nerve [4,5].

While varicella is usually a mild and self-limiting illness in immunocompetent children, it can pose significant health risks to vulnerable populations, including older adults, immunosuppressed individuals, and pregnant women [6,7]. One of the most frequent and debilitating complications of HZ is postherpetic neuralgia (PHN), a condition characterized by persistent neuropathic pain lasting beyond 90 days after the onset of the rash [7]. PHN affects approximately 10–30% of individuals with HZ, with the risk increasing significantly with age [7]. It can lead to chronic pain syndromes that diminish quality of life and require long-term pain management.

Other complications include secondary bacterial infections and ocular complications such as keratitis, uveitis, and retinitis, which can result in permanent vision impairment [7]. Neurological complications involving the central and peripheral nervous systems can lead to encephalitis, meningitis, myelitis, and facial paralysis [2,4]. Additionally, the involvement of the facial nerve can cause hearing loss.

In disseminated HZ, multiple organ systems, including the liver, lungs, and brain, can be affected. This severe form carries a high risk of mortality and requires aggressive antiviral therapy [3,7].

Maternal VZV infection during pregnancy increases the risk of severe complications for both the mother and the fetus. For the mother, complications may include encephalitis or pneumonitis, both of which can be life-threatening. For the fetus, maternal VZV infection can result in congenital varicella syndrome or severe neonatal complications, especially when the infection occurs near the time of delivery.

In the case of HZ, clinical manifestations during pregnancy resemble those seen in the general population. However, closer monitoring is essential to mitigate the risk of complications such as disseminated HZ or neurological involvement (Table 1) [6,7].

Although most cases of HZ can be managed on an outpatient basis, a subset of patients require hospitalization due to severe complications. The overall hospitalization rate for HZ in the general population ranges between 1 and 5%, with significantly higher rates in individuals over 65 and those who are immunosuppressed [7]. Factors that increase the likelihood of hospitalization include advanced age (especially >65 years), immunocompromised status (e.g., HIV, cancer, and transplant recipients), severe pain or PHN, neurological complications, and disseminated disease [6,7].

## 2. Epidemiological Aspects of VZV Infection in Pregnant Women

Primary VZV infection (chickenpox or varicella), is a common childhood disease. Research indicates that over 90% of individuals in the adult population are seropositive for VZV immunoglobulin G (IgG) antibodies [8]. Studies from Spain and France reported varicella immunity rates of 96.1% and 98.8% in pregnant women, respectively [9,10]. As a result, despite frequent exposure to chickenpox during pregnancy, particularly in women with young children, primary VZV infection during pregnancy remains rare, being estimated at 0.5–1.2 cases per 1000 pregnancies [11]. Since there are no studies, there is currently little available data on the incidence and prevalence of HZ in pregnant women. However, it is estimated that the incidence of HZ in pregnancy is approximately 1 in 20,000 [12].

The risk factors for primary VZV infection can be divided into host-related factors and external factors. Host-related factors primarily include a lack of previous immunity to the virus. External factors consist of close contact with infectious individuals, which can occur in household settings or in occupational environments that involve frequent interaction with children. Other external risk factors include living in crowded conditions and traveling to areas where the virus is endemic. Risk factors for HZ include immunosuppression during pregnancy, which can facilitate viral reactivation. Additional factors include advanced maternal age, chronic illnesses such as diabetes, rheumatoid arthritis, cardiovascular diseases, renal disease, systemic lupus erythematosus, and inflammatory bowel disease, physical or emotional stress, and trauma to a specific dermatome, which may trigger reactivation [13]. COVID-19 infection has also been identified as a risk factor for developing HZ [14]. However, current data suggest that COVID-19 vaccination does not significantly statistically reduce the risk of HZ [15]. Pregnant women with compromised immunity, such as those with HIV infection, those undergoing immunosuppressive therapy, or those receiving chemotherapy, are at a higher risk for both primary VZV infection and viral reactivation [16].

## 3. VZV Infection in Pregnant Women

The widespread transmission of VZV across Europe highlights the need for specialized attention to vulnerable populations, particularly pregnant women, due to the infection’s potential risks to both maternal and fetal health [6]. Among the most common maternal complications of primary VZV infection is varicella pneumonia, which affects up to 20% of pregnant women who develop chickenpox [5,17]. Severe complications, such as encephalitis and hepatitis, occur less frequently but are significant contributors to the increased morbidity and mortality rates associated with VZV in pregnancy compared to non-pregnant individuals [6,18,19,20].

Varicella pneumonia typically manifests within the first week after the onset of the chickenpox rash, presenting with fever, dry cough, shortness of breath, and mild hypoxemia [6,19]. Risk factors for developing varicella pneumonia include having more than 100 skin lesions, smoking, concurrent respiratory diseases, and immunosuppression [6,19,21].

Fetal complications resulting from maternal VZV infection depend on the gestational age at which the primary infection occurs [6,22]. Infections during the first trimester do not significantly increase the risk of miscarriage [23]. However, infections during the first and second trimesters may result in fetal varicella syndrome (FVS) or congenital varicella syndrome (CVS), with the highest risk (approximately 2%) occurring between 13 and 20 weeks of gestation. After 20 weeks, the incidence of FVS decreases significantly as the fetal immune system develops sufficient capacity to mount an immune response [6,21,24].

Infants born with CVS often experience severe health issues, including neurological and developmental disorders (microcephaly cortical atrophy and mental retardation), ocular abnormalities (microphthalmia, cataracts, and chorioretinitis), dermatomal scarring, limb hypoplasia, muscle underdevelopment, gastrointestinal and urinary tract anomalies (dysfunctional bowel and bladder sphincter), and recurrent aspiration pneumonia [5,25,26,27]. The prognosis for affected newborns is poor, with a mortality rate of approximately 30% within the first month of life [27]. Notably, maternal herpes zoster does not cause CVS [5,21].

Prenatal detection of CVS is primarily based on ultrasound evaluation. Pregnant women with primary VZV infection should be referred to a fetal medicine specialist for a comprehensive assessment and consultation, ideally between 16 and 20 weeks of gestation or approximately five weeks post-infection [28,29]. Key ultrasound findings indicative of CVS include limb deformities, microcephaly, hydrocephalus, soft tissue calcifications, polyhydramnios, and intrauterine growth restriction [30]. When abnormalities are identified, fetal MRI may offer further diagnostic insights [6].

Invasive procedures like amniocentesis can be used to detect VZV DNA and diagnose in utero varicella infection [31]. However, these tests have low specificity for identifying CVS and are not routinely recommended in clinical practice [28].

The risk of fetal abnormalities is lower when maternal varicella occurs between the 21st and 36th weeks of pregnancy, and the prognosis for affected infants is generally favorable. Such children may develop a mild form of HZ during early childhood, typically without long-term complications [6,22].

However, when maternal chickenpox occurs during the final four weeks of pregnancy, there is a significant risk of neonatal varicella. Neonatal infection may result from transplacental transmission, exposure to vesicular fluid during delivery, or respiratory droplets [6,18]. The risk of neonatal infection is mitigated if maternal chickenpox occurs early enough before delivery to allow for the transplacental transfer of protective anti-VZV antibodies [6].

Newborns exposed to maternal VZV shortly before delivery and two days after birth may develop fulminant neonatal infection. This is due to high intrauterine viral exposure without the protective benefits of maternal anti-VZV IgG antibodies, which would otherwise help mitigate disease severity. Despite this, the prognosis for these neonates is generally favorable. Advances in neonatal intensive care, antiviral therapies, and anti-VZV immunoglobulin administration have reduced the mortality rate of neonatal varicella to approximately 7% [6,21,32].

## 4. Management of Varicella Exposure in Pregnant Women

Different approaches to managing varicella exposure in pregnant women are listed in Table 2.

Screening for VZV immunity is recommended for all women of childbearing age before conception [33]. Women who test negative for anti-VZV antibodies should receive VZV vaccination before becoming pregnant. In the event of significant VZV exposure during pregnancy, management strategies should be tailored based on the woman’s immune status [16,33]. For individuals without a history of chickenpox or VZV vaccination, serological testing within the first two days post-exposure is essential to assess immunity. If the patient is found to be susceptible, administration of anti-VZV immunoglobulin is advised within 72–96 h, with efficacy extending up to 10 days post-exposure [33].

For exposures occurring beyond 10 days or when a pregnant woman develops a chickenpox rash, symptomatic treatment with antipyretic and antipruritic medications should be initiated [34]. This should be followed by antiviral therapy, specifically oral acyclovir, at a dose of 800 mg five times daily for seven days [17]. Antiviral treatment primarily benefits maternal health, reducing the duration of fever and severity of symptoms when initiated within 24 h of rash onset [34,36]. Acyclovir is particularly advantageous in cases of complications such as hemorrhagic or extensive rashes, respiratory symptoms, or seizures, and for individuals at higher risk, such as those who are immunocompromised, have chronic lung disease, or are in the late stages of pregnancy (≥36 weeks). In severe cases of maternal varicella, intravenous acyclovir combined with intensive supportive care is recommended [35].

VZV infections occurring within the last four weeks of pregnancy demand a different management approach due to the heightened risk of neonatal varicella [32,36]. Delivery during active maternal viremia accompanied by vesicular lesions increases the risk of complications such as maternal hemorrhage and coagulopathy, often caused by hepatitis or thrombocytopenia [35,36]. This also significantly elevates the likelihood of neonatal varicella [35,36].

Current guidelines recommend administering anti-VZV immunoglobulin to all neonates born to mothers who develop chickenpox from seven days before to seven days after delivery [35,37]. Although this intervention may not completely prevent neonatal infection, it significantly reduces symptom severity [35]. In cases where neonatal varicella occurs despite immunoglobulin prophylaxis, intravenous acyclovir therapy should be promptly initiated for the newborn [35].

## 5. Varicella Vaccination and Its Impact on Children and Newborns

Maternal antibodies against VZV are transferred to the fetus during pregnancy, providing transient immunity during the early months of life. However, the duration of this passive protection is limited, as maternal antibody levels decline rapidly, leaving infants increasingly susceptible to infection. Understanding the kinetics of this decline is essential for developing effective immunization strategies and preventive interventions.

Research has demonstrated a significant waning of maternal VZV antibodies in neonates. Bolotin et al. investigated the duration of seropositivity in newborns, revealing that most infants lose protective antibody levels within the first few months of life [38]. At one month, 32% of infants were susceptible to VZV infection, increasing to nearly 80% by three months, and by six months, all infants lacked detectable maternal antibodies [38]. The estimated half-life of passively acquired maternal anti-VZV antibodies is approximately six weeks, indicating that a considerable proportion of infants may no longer have protective immunity by three months of age [39].

The timing of maternal VZV infection relative to delivery is a critical determinant of neonatal immunity. Infants born to mothers who contract VZV between five days before and two days after delivery are at the greatest risk of severe neonatal varicella, as the maternal immune system does not have sufficient time to transfer protective antibodies [40]. In such cases, neonatal exposure to the virus without adequate antibody protection can result in severe outcomes [40].

Newborns lacking adequate maternal antibodies are particularly vulnerable to neonatal varicella, which can be life-threatening [36]. To mitigate this risk, the administration of varicella-zoster immunoglobulin to exposed neonates offers passive immunity and significantly reduces the severity of the disease [36]. As maternal antibodies wane completely by 6 to 12 months, timely vaccination against varicella becomes imperative to maintain immunity in infants [36].

Epidemiological data from the European Centre for Disease Prevention and Control (ECDC) highlight the significance of varicella as a childhood infection. Most cases in Europe occur in children under six years of age, with 52–78% of infections affecting this age group and 89–95.9% occurring before adolescence [5,41,42]. Varicella vaccination is strongly recommended for children and adults in close contact with high-risk individuals due to its proven safety and efficacy [5]. While both single-dose and two-dose vaccination regimens are effective, the two-dose schedule provides superior protection [5]. Germany serves as a model for vaccination implementation, having introduced universal varicella vaccination for children over 11 months of age in 2004 [5]. This initiative resulted in a substantial decline in varicella-related hospitalizations, with a 63% reduction in cases among children aged 0–4 years and a 38% reduction among those aged 5–9 years [5]. By 2012, Germany recorded an 84% reduction in varicella cases across all age groups [5].

## 6. Herpes Zoster in Pregnancy

Reactivation of VZV during pregnancy does not typically increase fetal mortality or result in congenital malformations. However, shingles and its associated complications can pose significant risks to maternal health [39,43]. Epidemiological studies demonstrate that HZ occurs less frequently in younger individuals compared to middle-aged and elderly populations [44]. Although routine assessments may not always be required for young patients with HZ, physicians should evaluate potential underlying immunosuppressive conditions in these cases [43,44,45]. Among healthy pregnant women presenting with HZ, screening for human immunodeficiency virus (HIV) and other causes of immunosuppression is recommended [46]. Notably, research suggests that women experience higher rates of HZ across all age groups, potentially due to gender-specific immune responses to latent viral infections [47]. Although rare, cases of HZ during pregnancy have been documented [46], but there is currently no evidence to support recommending HZ vaccination for pregnant women [48].

For neonates, maternal HZ typically poses no risk due to the transfer of protective maternal IgG antibodies [44]. However, in cases where the mother is immunocompromised, viremic dissemination of VZV may occur, as demonstrated in a case involving congenital varicella syndrome in a neonate born to a mother with disseminated zoster at 12 weeks of gestation [49]. Importantly, no instances of neonatal VZV infection have been reported following localized maternal HZ outbreaks [50].

When pregnant women contract varicella, there is an estimated 20% risk of primary VZV infection in utero, which may lead to neonatal or infantile HZ [51,52]. In the majority of cases, however, the disease course is uncomplicated [52,53]. Early-onset HZ in infants is thought to result from the reactivation of in utero acquired VZV, with the shorter latency period attributed to the immaturity of the infant’s cell-mediated immune response [53].

Particular attention is required when maternal genital HZ presents near term or at delivery. VZV, which remains latent in cranial and vertebral sensory ganglia, can reactivate in sacral ganglia, resulting in genital HZ [50]. Despite the protective role of maternally transmitted anti-VZV antibodies, the virus can be transmitted to the neonate via respiratory or mucocutaneous routes, posing a potential risk of neonatal VZV infection [50]. Although this risk is considered low, accurate diagnosis and appropriate management of maternal and neonatal care are critical.

Mariaggi et al. underscore the importance of monitoring genital herpes-like lesions during pregnancy through medical history, clinical examination, and molecular testing to confirm viral etiology and avoid misdiagnosis of atypical presentations [35]. Molecular diagnostics enable tailored obstetric and neonatal management. Following diagnosis, it is essential to assess the risk of VZV excretion in the genital area at the time of delivery. For preterm neonates lacking maternal antibodies, the risk of disseminated and severe VZV infection is significant. In such cases, the administration of specific VZV immunoglobulin is necessary. The authors also advocate for the development of evidence-based guidelines to optimize the management of HZ during pregnancy, particularly in cases involving genital lesions [35].

**Figure 1:**
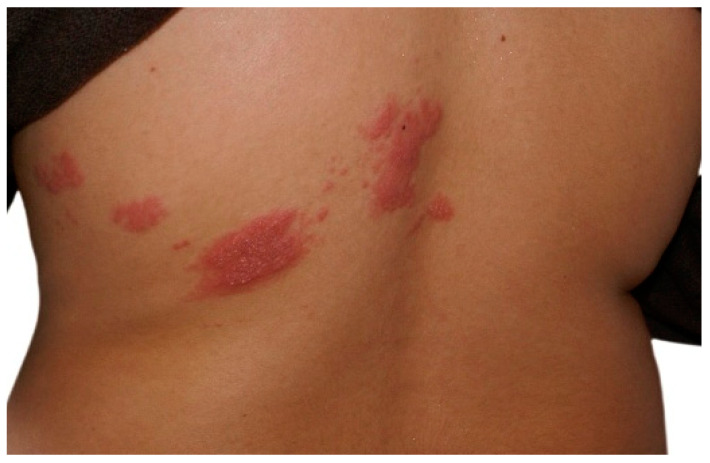
Typical clinical presentation of herpes zoster: asymmetrical dermatomal eruption on the posterior aspect of the trunk limited by the midline.

**Figure 2:**
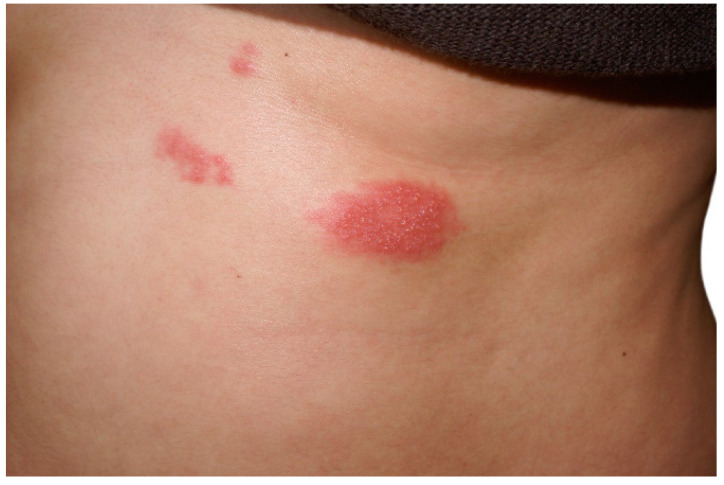
Detail: Erythematous plaque covered by multiple small, grouped vesicles on the posterior aspect of the trunk limited by the midline.

Clinically, HZ is diagnosed based on its characteristic presentation of a unilateral dermatomal distribution of grouped vesicles, often preceded by a prodromal phase of pain and segmental discomfort typical of zoster (Figure 1 and Figure 2). Differentiating HZ and varicella from other viral infections, such as herpes simplex virus, is essential [44]. Laboratory confirmation of VZV infection in pregnant women and neonates is often required [54]. Diagnostic methods include direct virus detection in cell cultures, polymerase chain reaction (PCR), and serological tests. Immunofluorescence and enzyme-linked immunosorbent assay (ELISA) can identify VZV-specific IgM, IgG, and IgA antibodies. While anti-VZV IgG levels may increase spontaneously or due to cross-reactivity with the herpes simplex virus, the presence of IgM and IgA antibodies reliably indicates VZV reactivation, regardless of clinical symptoms [54].

## 7. Management of HZ in Pregnancy

HZ is generally not associated with adverse pregnancy outcomes; however, it carries significant implications for maternal health due to the potential severity and debilitating nature of its complications [33,55]. Prompt antiviral treatment is recommended for pregnant women with HZ, as it is believed to expedite the resolution of skin lesions and reduce both the duration and intensity of pain [33,56]. Given the limited evidence suggesting an increased risk of complications during pregnancy, some experts advocate for treatment only in cases of severe HZ outbreaks or acute neuritis [29].

Currently, three FDA-approved antiviral medications—acyclovir, valacyclovir, and famciclovir—are used for HZ treatment. While data on their use during pregnancy are limited, their effectiveness in managing herpes simplex virus infections and varicella pneumonia supports their utility in HZ [33,57]. Available evidence suggests minimal fetal risk associated with prenatal antiviral use [45,58]. A large national cohort study conducted by Pasternak and Hviid in 2010 found no association between first-trimester exposure to antivirals (acyclovir, valacyclovir, or famciclovir) and major birth defects [59].

The timing of treatment is critical for maximizing antiviral efficacy, with initiation ideally occurring within 48–72 h of skin lesion appearance [33]. In most cases, oral antiviral therapy suffices, with acyclovir recommended as the first-line treatment at a dosage of 800 mg five times daily for 7–10 days. However, certain clinical scenarios necessitate intravenous antiviral therapy (Table 3) [33].

### 7.1. Complications of HZ

Complications of HZ include post-herpetic neuralgia (PHN), secondary bacterial infections, ophthalmologic issues, peripheral facial nerve palsy, and disseminated HZ, all of which require tailored management strategies.

#### 7.1.1. Post-Herpetic Neuralgia (PHN)

PHN is a prominent complication, characterized by persistent pain lasting 90 days or more after the initial rash [44]. Effective management involves a range of therapeutic options, including acetaminophen, non-steroidal anti-inflammatory drugs (NSAIDs), opioids, antidepressants, gabapentin, pregabalin, and topical analgesics [44,61]. Although combinations of analgesics and neuroactive agents are standard in non-pregnant patients, HZ management during pregnancy presents unique challenges [44,62]. Oral acetaminophen is the preferred first-line treatment for pain relief due to its safety profile throughout pregnancy [63]. NSAIDs may also be considered but should be limited to the first and second trimesters to avoid complications such as premature ductal closure in later pregnancy [64]. Additional therapies include lidocaine patches, capsaicin cream, sympathetic blockade with local anesthetics, and transcutaneous electrical nerve stimulation (TENS) (Table 4) [46,63,64,65]. For severe acute pain, opioid analgesics may be necessary, though careful management is required to minimize dosage and duration due to risks of maternal dependency and neonatal outcomes, such as neonatal abstinence syndrome [63,65]. Evidence regarding opioid use during pregnancy is mixed, and the general recommendation is to use the lowest effective dose for the shortest possible period [64].

#### 7.1.2. Ophthalmologic and Neurological Complications

Ocular complications, such as zoster ophthalmicus, are among the most frequent and arise from VZV reactivation in the ophthalmic division (V1) of the trigeminal nerve [44]. Pregnant women with HZ-related eye involvement should receive immediate ophthalmologic evaluation. Neurological complications, such as zoster oticus, necessitate referral to an ENT specialist, particularly when associated with geniculate ganglion involvement, which may result in peripheral facial nerve palsy or Ramsay-Hunt syndrome [61].

#### 7.1.3. Secondary Bacterial Infections

HZ lesions may develop secondary bacterial infections, typically caused by streptococcal or staphylococcal species [54]. Clinical signs of infection, such as warmth, erythema, or purulent discharge, require prompt medical attention. If bacterial infection is suspected, appropriate antibiotic therapy should be initiated alongside antiviral treatment to prevent complications such as hemorrhagic vesicles, ulcerations, or persistent lesions [54].

#### 7.1.4. Disseminated Herpes Zoster

Disseminated HZ is primarily observed in immunocompromised individuals and involves vesicular lesions extending beyond the initial dermatome, with potential multiorgan involvement. This severe form of HZ can lead to pneumonia, hepatitis, encephalitis, coagulopathy, thrombocytopenia, or hemorrhagic lesions, with a mortality rate of 5–10% despite antiviral treatment [60].

### 7.2. Prophylaxis and Prevention

Neither the varicella vaccine nor the zoster vaccine is recommended for pregnant women or those planning pregnancy within 30 days of vaccination. Pregnant women lacking VZV immunity should receive the first vaccine dose postpartum, typically before hospital discharge, to protect their newborns through herd immunity [44]. In cases of uncertain or negative varicella exposure during pregnancy, anti-VZV IgG antibody levels should be promptly tested. If serology results are indeterminate, negative, delayed, or unavailable following confirmed high-risk exposure, varicella-zoster immune globulin (VZIG) should be administered within 72–96 h at a dose of 125 U per 10 kg of body weight [44]. Vaccinated pregnant women testing VZV IgG-negative should be managed as seronegative for varicella. Additionally, neonates should receive VZIG if maternal HZ onset occurs between five days before and two days after delivery [44].

To prevent the reactivation of HZ, two vaccines have been approved by the U.S. Food and Drug Administration (FDA): Zostavax and Shingrix (RZV) [58,66]. Zostavax is a live attenuated vaccine that was developed to reduce the risk of shingles and its complications. However, due to waning immunity over time and the availability of a more effective alternative, its use has declined in recent years. Shingrix (RZV) is a recombinant, adjuvanted vaccine that provides stronger and longer-lasting immunity. It is the preferred choice for shingles prevention and is recommended for adults aged 50 years and older, as well as individuals 18 years and older who are immunocompromised. The vaccine consists of two doses administered 2 to 6 months apart, or in certain cases, at a shorter interval of 1 to 2 months for immunocompromised individuals [48]. Regarding pregnancy, there are currently no established guidelines for administering RZV. Since it is a non-live vaccine, the theoretical risk is lower compared to live vaccines, but clinical safety data for its use during pregnancy are still limited [66]. As a precaution, it is generally recommended to delay vaccination until after pregnancy, unless the potential benefits outweigh the risks in specific cases. Healthcare providers should assess individual patient circumstances before making a recommendation.

## 8. Conclusions

VZV infection during pregnancy poses a substantial risk of severe illness for both the mother and newborn. However, herpes zoster during pregnancy does not appear to increase the likelihood of complications such as maternal pneumonia, birth defects, or other perinatal issues. Although HZ can be concerning, clinical attention should primarily focus on the health and well-being of the mother.

For pregnant women with genital HZ near delivery, particularly in preterm births, the risk of disseminated neonatal VZV infection is elevated. In these cases, specific VZV immunoglobulin is critical to prevent severe outcomes. Studies, including cohort and registry analyses, have confirmed the safety of acyclovir use during pregnancy, with no evidence of increased risk for major birth defects. Pain management remains a priority in treating pregnant women with HZ, and the administration of varicella-zoster immune globulin is recommended in cases of unknown serologic status or confirmed high-risk exposure. Pregnant patients should also be counseled on the potential for HZ to cause primary infection in individuals without prior VZV exposure or vaccination.

## Figures and Tables

**Table 1:** Clinical presentations of HZ.

Classic Herpes Zoster	- a unilateral, painful, vesicular eruption developed in a single dermatome
Disseminated Herpes Zoster	- widespread vesicular lesions beyond the primary dermatome, resembling varicella
Ophthalmic Herpes Zoster	- involvement of the ophthalmic branch of the trigeminal nerve - vesicular rash on the forehead, eyelids, or tip of the nose (Hutchinson’s sign) - conjunctivitis, keratitis, uveitis, or even vision loss in severe cases
Herpes Zoster Oticus (Ramsay Hunt Syndrome)	- involvement of the facial nerve - ear pain - vesicles on the auricle or in the auditory canal - can lead to peripheral facial nerve palsy
Atypical Herpes Zoster (zoster sine herpete)	- neurological manifestations in the absence of the typical skin rash that follows a specific dermatome

**Table 2:** Different approaches to managing varicella exposure in pregnant women.

Approach	Indication	Contraindications	Advantages	Disadvantages	References
Screening and Vaccination (Pre-Pregnancy)	All women of childbearing age without documented immunity to VZV.	Pregnancy (live attenuated vaccine contraindicated).	Prevents varicella during pregnancy; long-term immunity.	Not applicable during pregnancy; requires pre-pregnancy planning.	[33]
Anti-VZV Immunoglobulin (Post-Exposure)	Susceptible pregnant women (no history of chickenpox or VZV vaccination) within 10 days of exposure.	Known immunity to VZV.	Reduces risk of severe maternal varicella and congenital varicella syndrome (CVS); safe during pregnancy.	Must be administered within a specific timeframe (72–96 h post-exposure for maximum efficacy).	[16,33]
Symptomatic Treatment + Oral Acyclovir	Pregnant women with active chickenpox rash or VZV exposure >10 days earlier.	Severe renal impairment without adjustment or hypersensitivity to acyclovir.	Reduces fever duration and symptom severity when started early (within 24 h of rash onset).	Limited efficacy if started late; primarily benefits the mother; potential adverse effects with prolonged use.	[34,35]
Intravenous Acyclovir + Supportive Care	Severe maternal Varicella (E.G., Respiratory symptoms, complications, immunocompromised, or late pregnancy).	Hypersensitivity to acyclovir; cautious use in renal impairment.	Effective in treating complications; reduces maternal mortality.	Requires hospitalization; potential nephrotoxicity.	[34]
Management During Last 4 Weeks of Pregnancy	Pregnant women with active varicella rash within 7 days before or after delivery.	Nonspecific unless maternal contraindications exist.	Reduces neonatal varicella severity when anti-VZV immunoglobulin is given to newborns.	May not fully prevent neonatal varicella; requires close monitoring and additional treatment with IV acyclovir.	[32,35,36,37]
Intravenous Acyclovir for Neonates	Neonates who develop varicella despite immunoglobulin prophylaxis.	Hypersensitivity to acyclovir.	Reduces severity and complications of neonatal varicella.	Requires hospitalization; limited efficacy in preventing long-term complications if started late.	[35,37]

**Table 3:** Indications for intravenous antiviral therapy for HZ [34].

Indication	Advantages	Contraindications	Side Effects	Disadvantages	References
Pneumonitis	Effective in treating severe respiratory complications of disseminated HZ.	Severe renal impairment (dose adjustment).	Nephrotoxicity, nausea, vomiting, diarrhea, rash.	Requires hospitalization; close monitoring for renal toxicity is needed.	[33,60]
Neurologic complications (meningitis, encephalitis, myelitis)	Improvement of neurologic deficits.	Severe renal impairment (dose adjustment).	Nephrotoxicity, nausea, vomiting, diarrhea, rash.	Requires hospitalization; close monitoring for renal toxicity is needed.	[33,50]
Ocular involvement	Prevents progression to vision-threatening complications (E.G., Keratitis, retinitis).	Nonspecific; caution in hypersensitivity.	Nephrotoxicity, headache, dizziness.	Requires immediate ophthalmologic evaluation alongside antiviral therapy.	[44,60]
Ramsay Hunt syndrome	In severe cases, it prevents progression toward hearing loss.	Nonspecific; caution in hypersensitivity.	Nephrotoxicity, headache, dizziness.	Requires hospitalization.	[7]
Hemorrhagic lesions	Reduces viral replication and risk of systemic spread in immunocompromised patients.	Hypersensitivity to acyclovir.	Renal dysfunction, inflammation at infusion site.	Limited efficacy if started late (>72 h after rash onset).	[33,56]
Mucosal involvement	Treats severe mucosal damage and reduces discomfort and risk of secondary infections.	Severe dehydration or fluid imbalance.	Gastrointestinal symptoms, hematologic effects.	Limited studies available on outcomes specific to mucosal HZ.	[33,56,60]
Involvement of cranial nerves	Reduces the risk of complications, such as Ramsay-Hunt syndrome, peripheral nerve palsy, or hearing loss.	Caution in patients with renal or hepatic impairment.	Nephrotoxicity, elevated liver enzymes.	Requires adjunct care with ENT or neurologic specialists.	[33,60]
Persistent symptoms after 6 days of systemic therapy	Provides additional efficacy for unresolved or worsening symptoms.	Contraindicated in severe hypersensitivity.	Fatigue, rash, phlebitis, and neurotoxicity in rare cases.	Delayed initiation reduces effectiveness; risk of developing antiviral resistance in prolonged therapy.	[33,57]

**Table 4:** Therapeutic options for optimal management of post-herpetic neuralgia during pregnancy.

Therapeutic Options	Advantages	Contraindications	Side Effects	Disadvantages	References
Oral Acetaminophen	Safe across all pregnancy stages; effective for mild to moderate pain.	Severe hepatic impairment.	Hepatotoxicity with overdose, nausea, and rash.	Limited efficacy for severe pain; requires frequent dosing for sustained effect.	[44,63]
NSAIDs (first and second trimesters)	Effective for inflammation-related pain; well-studied safety profile in early pregnancy.	Third trimester (risk of premature ductal closure); hypersensitivity.	Gastrointestinal discomfort, renal effects.	Contraindicated in the third trimester due to risks of fetal complications, including oligohydramnios.	[44,64]
Lidocaine Patches	Provides localized pain relief without systemic absorption; minimal fetal exposure.	Hypersensitivity to lidocaine.	Skin irritation, erythema, or itching at the application site.	Limited efficacy for deep or widespread pain; requires regular application and monitoring.	[44,64]
Capsaicin Cream	Reduces pain through desensitization of nociceptive neurons; low systemic absorption.	Hypersensitivity to capsaicin or skin lesions.	Local burning, stinging, or erythema during initial use.	Initial application discomfort may deter adherence; not suitable for open or irritated skin.	[64]
Sympathetic Blockade	Provides immediate relief for severe, localized pain; can reduce reliance on systemic analgesics.	Coagulopathy, infection at injection site.	Rare complications: nerve injury or infection.	Requires specialist care; limited availability in some settings; invasive procedure with associated risks.	[44,64]
Transcutaneous Electrical Nerve Stimulation (TENS)	Non-invasive, drug-free pain management; no known teratogenic effects.	Implanted pacemaker or other electronic devices.	Skin irritation at electrode sites, and discomfort during application.	Variable efficacy; requires patient adherence and proper device use; optimal settings may need adjustment over time.	[44,64]
Opioid Analgesics	Effective for severe pain when other treatments fail; short-term use is recommended.	Respiratory depression in mother or fetus; history of substance misuse.	Drowsiness, constipation, and potential for dependency.	Requires careful dosing and monitoring to avoid maternal dependency and neonatal abstinence syndrome.	[44,63,65]

## Data Availability

Data are contained within the article.
